# Safety and efficacy of sonography-guided PCNL under local versus general or spinal-epidural anesthesia: A meta-analysis

**DOI:** 10.1097/MD.0000000000044021

**Published:** 2025-08-22

**Authors:** Yong Cai, Junbo Liu, Yong Chen, Qiang Liu, Fei Lai

**Affiliations:** a Department of Urology, Chengdu Second People’s Hospital, Chengdu, Sichuan Province, P.R. China.

**Keywords:** calculi, combined spinal-epidural anesthesia, general anesthesia, local infiltration anesthesia, meta-analysis, Percutaneous nephrolithotomy

## Abstract

**Background:**

To conduct a comprehensive meta-analysis of existing evidence to compare the safety and efficacy of sonography-guided percutaneous nephrolithotomy (PCNL) under local infiltration anesthesia and both general anesthesia (GA) and combined spinal-epidural anesthesia for upper urinary calculi.

**Methods:**

We conducted a systematic literature search in the EMBASE, MEDLINE, Cochrane databases, China National Knowledge Infrastructure, Chinese Biomedical Literature Database, and Google Scholar to identify relevant studies published in English or Chinese up to March 2024. Literature reviewed included randomized and nonrandomized studies. The subject in the management of PCNL under local and GA of studies being patients who had a disease of upper urinary calculi were selected. The odds ratio and mean difference with 95% confidence intervals (CI) were calculated using fixed- or random-effects models. Two reviewers independently assessed the quality of all included studies, and the RevMan 5.3 and Stata 12.0 software was used to analyze the included studies.

**Results:**

Seven studies with 899 patients showed that, comparing with PCNL under GA or combined spinal-epidural anesthesia, PCNL under local infiltration anesthesia offered a significantly shorter operative time (MD = −18.91, 95% CI: −26.47 to −11.35, *P* < .00001, *I*^2^ = 96%), lower hospitalization expenses (MD = −4097.43,95% CI: −4203.26 to 3991.59, *P* < .00001, *I*^2^ = 0%), lower complication rate (OR = 0.49, 95% CI: 0.33–0.73, *P* = .0005, *I*^2^ = 0%), shorter postoperative hospital stay (MD = −1.85, 95% CI: −2.47 to 1.24, *P* = .001, *I*^2^ = 85%). But no statistical significant difference was found in stone-free rate between PCNL under local infiltration anesthesia and GA or combined spinal-epidural anesthesia (OR = 1.67, 95% CI: 0.54–5.15, *P* = .37, *I*^2^ = 41%).

**Conclusion:**

This meta-analysis compared efficacy and safety of PCNL under local infiltration anesthesia and both GA and combined spinal-epidural anesthesia for upper urinary calculi. Both of them were safe and effective for patients of upper urinary calculi. PCNL under local anesthesia offered a shorter operative time, lower hospitalization expenses, lower complication rate and shorter postoperative hospital stay for upper urinary calculi.

## 1. Introduction

Percutaneous nephrolithotomy (PCNL) is an elegant procedure to treat the patients of urinary calculi that has first been described in 1976.^[1]^ For multiple or single large renal stones, nowadays this approach is the first choice of therapy, and it is also indicated for upper ureteral calculi (UUC).^[2,3]^ The procedure of PCNL caused serious problems because of bleeding, infection, and other complications in the initial stage of its development^[4]^ Due to improvement in experience and technique, the efficacy and safety of PCNL surgery has greatly improved, although there are still concerns about the risk of its complications.^[5]^ As a general rule, this PCNL surgery is mainly performed under general anesthesia (PCNL-GA) or combined spinal-epidural anesthesia (PCNL-CSEA) because of light pain, convenient for intraoperative management. However, PCNL-GA or PCNL-CSEA has some shortcomings and limitations, such as high requirements for patients’ lung function, many adverse reactions of anesthesia, higher cost and so on. Recently, with the emergence of rapid rehabilitation theory, some urologists are interested in performing PCNL under local infiltration anesthesia (PCNL-LIA), and there have been a few published reports on conducting PCNL-LIA.^[6,7]^ PCNL-LIA has the characteristics of rapid recovery, low cost, and low requirements for patients’ cardiopulmonary function. Of course, surgical experience and the application of ultrasound technology are the key to the success of surgery, which also highlights the importance of operator training and the growing adoption of ultrasound guidance in PCNL procedures.^[8]^

However, so far there is no meta-analysis comparing safety and efficacy of sonography-guided PCNL-LIA and PCNL-GA or PCNL-CSEA for UUC. Therefore, the present study focused on a comprehensive meta-analysis of existing evidence to quantify and compare the safety and efficacy of sonography-guided PCNL-LIA and PCNL-GA or PCNL-CSEA for upper urinary calculi. The primary outcomes included stone-free rate (SFR), operative time, hospitalization expenses, complication rate and postoperative hospital.

## 2. Methods

The meta-analysis was conducted and reported according to Preferred Reporting Items for Systematic Reviews and Meta-analysis (PRISMA) statement.^[9]^

### 2.1. Data sources and searches

We conducted a systematic literature search in the China National Knowledge Infrastructure, Chinese Biomedical Literature Database (CBM), PubMed, EMBASE, MEDLINE, Cochrane databases and Google Scholar to identify relevant studies which reported safety and efficacy of PCNL-LIA and PCNL-GA or PCNL-CSEA for calculi in upper urinary tract published in English or Chinese up to March 2024. The Medical Subject Heading (MeSH) terms and/or key words and/or free words were (PCNL OR percutaneous nephrolithotomy) AND (local infiltration anesthesia OR local anesthesia OR LIA OR LA) AND (GA OR CSEA) AND (upper urinary calculi or UUC). Then an additional manual searches using the reference lists from key studies to retrieve other papers relevant to our topic was performed. And we also contacted corresponding authors to obtain some missing data from selected studies.

### 2.2. Study selection

Two reviewers (LG and LY) reviewed all the full texts of the identified studies. The studies that met the following inclusion criteria were included in the meta-analysis: 1. Due to the limited number of RCTs related to the content of this article, we included 2 different types of studies. The study had a randomized control design or a retrospective case control design; 2. The subject in the management of PCNL-LIA and PCNL-GA or PCNL-CSEA for calculi in upper urinary tract; 3. Studies comparing PCNL-LIA to either PCNL-GA or PCNL-CSEA were included. And the evaluated points including SFR, hospitalization expenses, operative time, postoperative hospital stay, complication rate, must be 2 aspects at least in the studies. 4. The language of the study must be published in English or Chinese. All studies that did not meet the above criteria were excluded.

### 2.3. Data extraction and quality assessment

A standardized data extraction form collecting information on the year of study period, country, study design, number of cases and controls, age, sex, the levels of evidence (LE), study quality SFR, operative time, postoperative hospital stay, hospitalization expenses and complication rate, was used to extract data. The LE for all included studies were estimated independently by 2 reviewers (QL and YC) according to the criteria provided by the Oxford Centre for Evidence Based Medicine.^[10]^ Two independent reviewers (QL and YC) appraised and determined the methodological quality of all included study according to the criteria of the Newcastle-Ottawa Scale (NOS) for nonrandomized controlled trials (RCTs)^[11]^ and the Jadad Scale for RCTs.^[12]^ The RevMan 5.3 software’s system-provided evaluation standards was also used to assess the risk of bias of all included studies. We would found a solution by discussing or inviting the third researcher to assist in. And we defined the Jadad scores as > 2 being high methodological quality and ≤ 2 being low quality. We also defined the NOS scores as 6 to 9 being high methodological quality and < 6 being low quality. The NOS quality scores and the Jadad scores were presented did not influence decisions to pool studies in the meta-analysis, it was only as part of descriptive summaries for each study. We also used the Cochrane Collaboration’s tool for assessing risk of bias to assess each study. The evaluate results was presented by using “Low bias,” “Uncertain” or “High bias.” When there were some different opinions of the 2 reviewers for a study.

### 2.4. Data synthesis and meta-analysis

The results of all data analysis are presented in forest plots. The heterogeneity is classified as high (*I*^2^ > 50%) and low (*I*^2^ ≤ 50%). We use the random or the fixed effect model in our meta-analysis according to that the homogeneity is high or low. We will conduct subgroup analysis or sensitivity analysis, if high heterogeneity (*I*^2^ > 50) is still found. The odds ratio (OR) and mean difference with 95% confidence intervals (CI) were calculated using fixed- or random-effects models. The funnel plot was used to estimate the publication bias. For all statistical analyses, a 2-sided *P* < .05 was considered statistically significant. Review Manager Software (RevMan v.5.3, Cochrane Collaboration, Oxford, UK) and STATA 12.0 are used to conduct the data analysis.

## 3. Results

### 3.1. Literature search and study election

**Figure 1.**
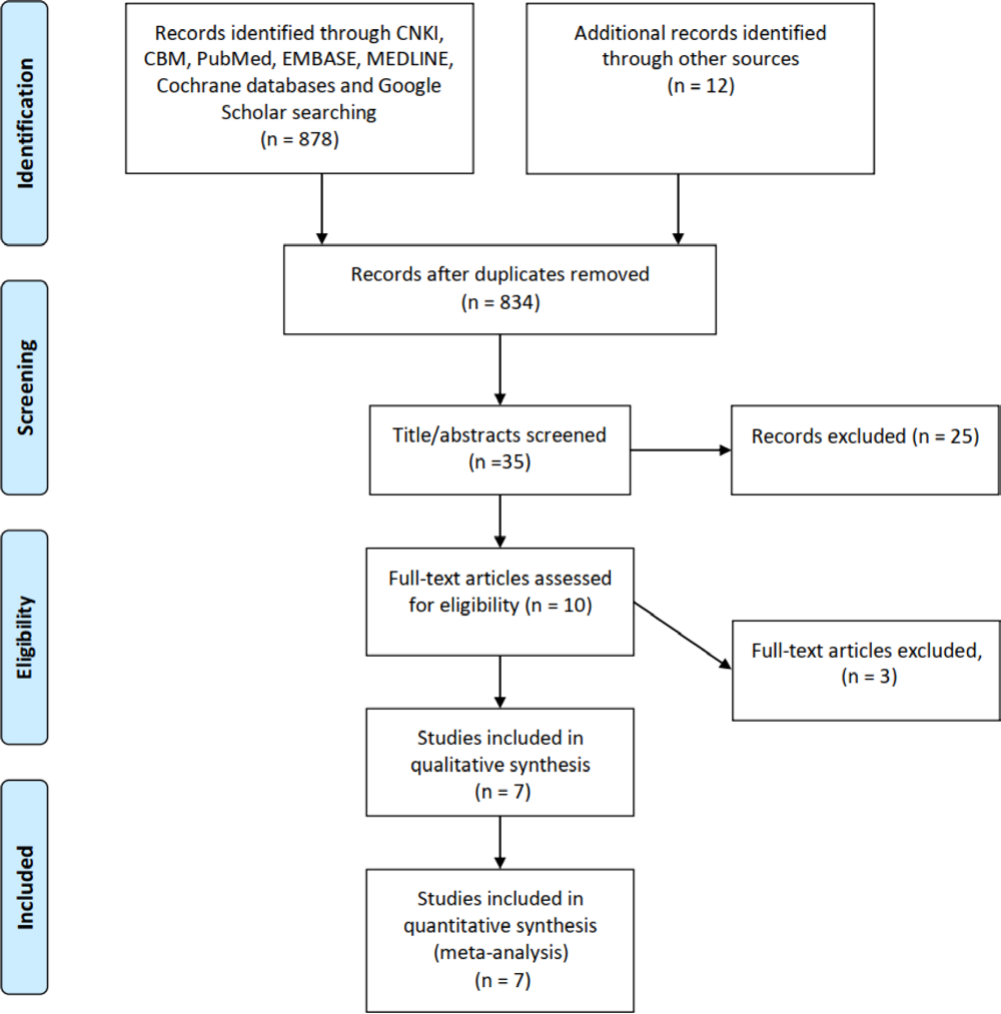
Flowchart for records selection process of the meta-analysis (according to PRISMA template: Moher D, Liberati A, Tetzlaff J, Altman DG, The PRISMA Group (2009). Preferred reporting items for systematic reviews and meta-analyses: the PRISMA statement. *PLoS Med* 6(7):e1000097. doi:10.1371/journal.pmed1000097).

**Figure 2.**
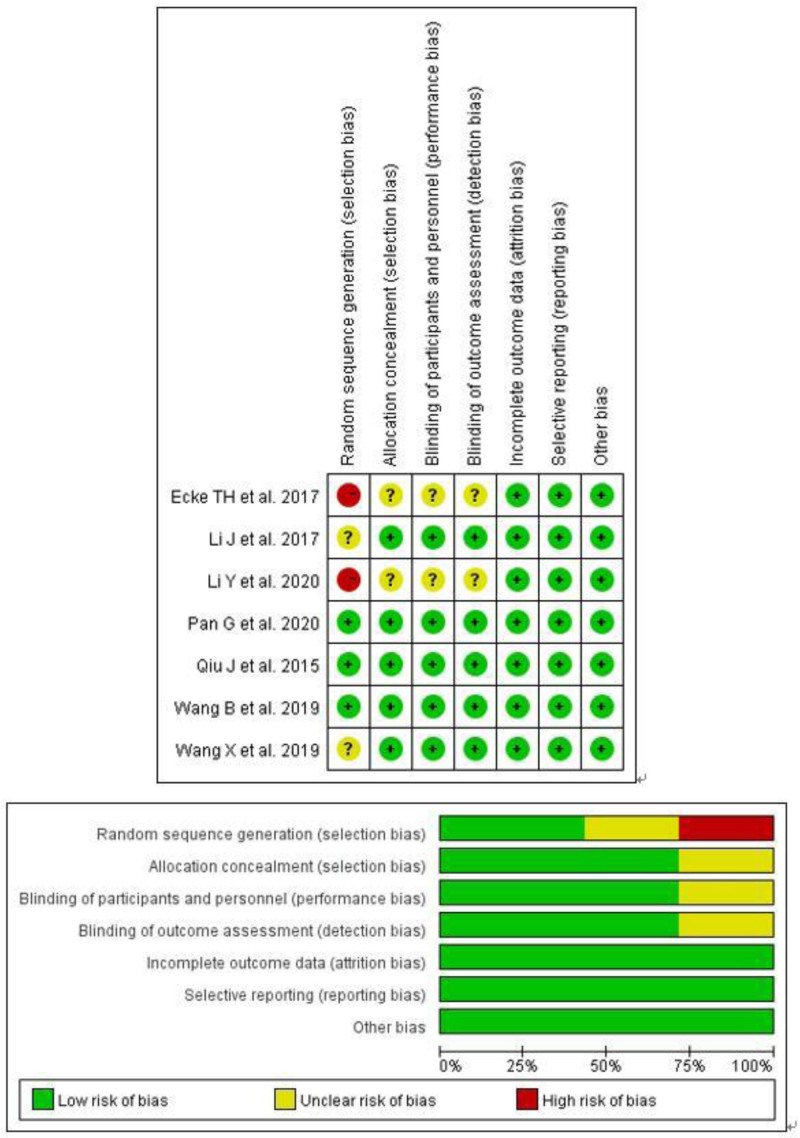
Risk of bias graph and risk of bias summary.

A PRISMA^[9]^ flow chart of screening and selection results is shown in Figure 1. After a systematic literature search, we retrieved 878 extracts and obtained 12 additional citations by other sources. From 35 studies initially identified, 10 were considered potentially suitable. After a full-text review, 7 studies,^[13–19]^ including 5 RCTs^[15–19]^ and 2 retrospective case controls,^[13,14]^ with 899 upper urinary calculi patients met the inclusion criteria and were included in the final analysis. NOS quality scores of 2 the retrospective case control were 7 scores, all of them were high quality. The Jadad scores of all the RCTs were 3, all of them also were high quality. Figure 2 shows the risk of bias for all the 7 studies^[13–19]^ assessed and summary results for the domains. The main sources of bias was selection bias. NOS quality scores and Jadad scores are showed in Table 1.

**Table 1:** Characteristics and quality assessment of the studies included in the meta-analysis.

Studies	Country	Study period (year)	Study design	Treatment	Case, n	LE	Study quality
Ecke et al 2017^[13]^	Germany	2003–2012	Retrospective case control	PCNL-LIA	226	3b	7*
				PCNL-GA	213		
Wang et al 2019^[18]^	China	2017–2018	RCT	PCNL-LIA	50	2b	3†
				PCNL-GA	50		
Li et al 2020^[14]^	China	2019–2020	Retrospective case control	PCNL-LIA	42	3b	7*
				PCNL-GA	42		
Wang et al 2019^[19]^	China	2017	RCT	PCNL-LIA	16	2b	3†
				PCNL-GA	20		
Pan et al 2020^[16]^	China	2018–2020	RCT	PCNL-LIA	30	2b	3†
				PCNL-CSEA	30		
Li et al 2017^[15]^	China	2012–2016	RCT	PCNL-LIA	50	2b	3†
				PCNL-GA	70		
Qiu et al 2015^[17]^	China	2010–2012	RCT	PCNL-LIA	30	2b	3†
				PCNL-CSEA	30		

“-” = No specific figures but without significant difference.

LE = levels of evidence, PCNL-CSEA = percutaneous nephrolithotomy under combined spinal-epidural anesthesia, PCNL-GA = percutaneous nephrolithotomy under general anesthesia, PCNL-LIA = percutaneous nephrolithotomy under local infiltration anesthesia, RCT = randomized controlled trial.

* Using Newcastle-Ottawa scale (score from 0 to 9).

† Jadad scale (score from 0 to 5).

A total of 899 patients were compared in our meta-analysis. In all included studies, there were 4 studies^[14,16–18]^ comparing SFR, 6 studies^[14–19]^ comparing operative time, 3 studies^[14,16,18]^ comparing hospitalization expenses, 4 studies^[13,14,17,18]^ comparing complication rate and 3 studies^[14,15,18]^ comparing postoperative hospital stay. Table 1 provides a description of the 7 studies^[13–19]^ which were published between 2015 and 2020. Most studies showed the preoperative demographic characteristics such as patients’ mean age, sex ratio, in addition, it included regions, study period and so on. Table 2 provides the summary of baseline patient characteristics and the relevant evaluating indexes of included studies.

**Table 2:** Summary of baseline patient characteristics and the relevant evaluating indexes of included studies.

Studies	Treatment	Age (year)	Case, n	Sex (male/female)	Operation time (min)	Postoperative hospital stay (d)	Hospitalization expenses (RMB)	SFR (%)	Complication rate (%)
Ecke et al 2017^[13]^	PCNL-LIA	63 (27–90)	226	129/ 97	39.5 (13–137)	–	–	–	31 (13.7%)
	PCNL-GA	56 (14–84)	213	118/ 95	48 (9–250)	–	–	–	45 (21.1%)
Wang et al 2019^[18]^	PCNL-LIA	31.9 (29–56)	50	25/ 25	50.16 ± 2.65	5.62 ± 0.63	–	48 (96%)	6 (12%)
	PCNL-GA	32.2 (28–55)	50	25/ 25	66.89 ± 3.26	7.56 ± 0.55	–	48 (96%)	13 (26%)
Li et al 2020^[14]^	PCNL-LIA	67 ± 1.6 (62–68)	42	31/ 11	41.0 ± 4.7	5.6 ± 1.5	15 016.3 ± 1728.3	40 (95.2%)	3 (7.1%)
	PCNL-GA	65 ± 1.1 (63–70)	42	26/ 16	73.0 ± 6.4	8.1 ± 1.4	18 907.3 ± 2228.3	39 (92.9%)	8 (19%)
Wang et al 2019^[19]^	PCNL-LIA	41–77	16	11/ 5	100 ± 7.7	–	17115.0 ± 918.4	–	–
	PCNL-GA	38–82	20	9/ 11	120 ± 9.0	–	21247.8 ± 1384.2	–	–
Pan et al 2020^[16]^	PCNL-LIA	56.39 ± 3.95	30	14/ 16	63.96 ± 14.86	–	–	29 (96.67%)	–
	PCNL-CSEA	48.82 ± 3.91	30	17/ 13	85.32 ± 18.95	–	–	21 (70%)	–
Li et al 2017^[15]^	PCNL-LIA	45.2 (18–72)	50	40/ 10	53.4 ± 12.1	7.8 ± 1.2	9220 ± 244	–	–
	PCNL-GA	46.5 (19–74)	70	56/ 14	66.5 ± 13.2	9.0 ± 1.2	13320 ± 358	–	–
Qiu et al 2015^[17]^	PCNL-LIA	–	30	30	87 ± 28	–	–	22 (73.3%)	5 (16.7%)
	PCNL-CSEA		30	30	93 ± 24	–	–	23 (76.6%)	12 (40%)

“-” = No specific figures but without significant difference.

PCNL-CSEA = percutaneous nephrolithotomy under combined spinal-epidural anesthesia, PCNL-GA = percutaneous nephrolithotomy under general anesthesia, PCNL-LIA = percutaneous nephrolithotomy under local infiltration anesthesia, RCT = randomized controlled trial, SFR = stone-free rate.

### 3.2. Results of meta-analyses

#### 3.2.1. Stone-free rate

**Figure 3.**
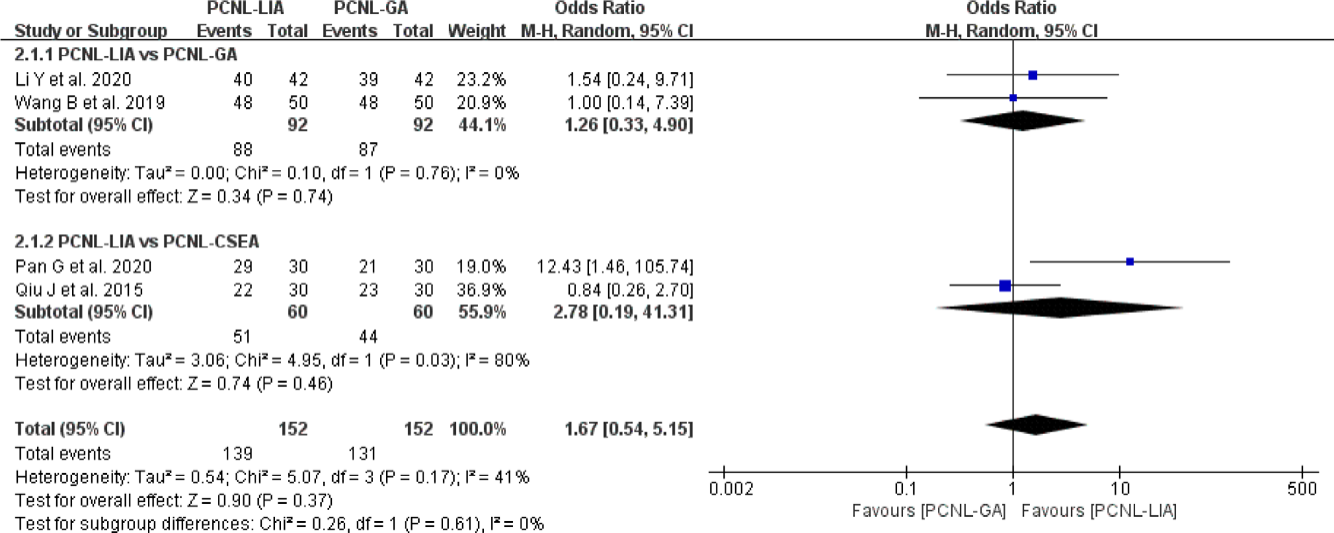
Forest plot comparing SFR between PCNL-LIA and PCNL-GA or PCNL-CSEA. PCNL-CSEA = percutaneous nephrolithotomy under combined spinal-epidural anesthesia, PCNL-GA = percutaneous nephrolithotomy under general anesthesia, PCNL-LIA = percutaneous nephrolithotomy under local infiltration anesthesia, SFR = stone-free rate.

In all included studies, there were 4 studies^[14,16–18]^ comparing SFR. In terms of the SFR, there was no statistical significant difference of SFR found between PCNL-LIA and PCNL-GA or PCNL-CSEA for UUC. (OR = 1.67, 95% CI: 0.54–5.15, *P* = .37, *I*^2^ = 41%) (Fig. 3).

#### 3.2.2. Operative time

**Figure 4.**
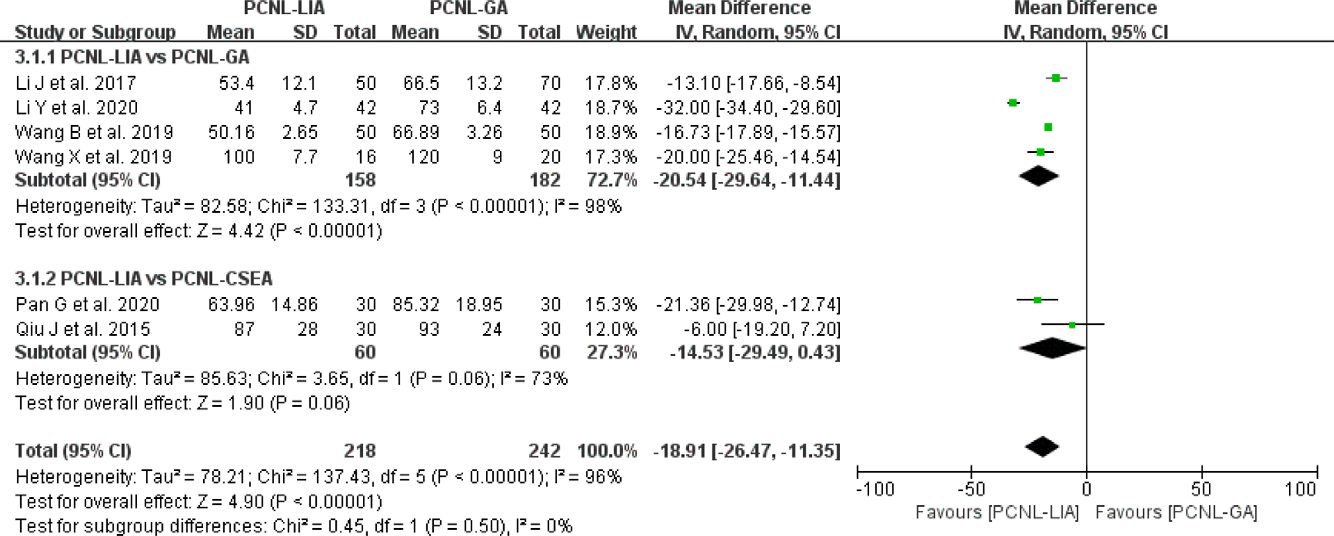
Forest plot comparing operative time between PCNL-LIA and PCNL-GA or PCNL-CSEA. PCNL-CSEA = percutaneous nephrolithotomy under combined spinal-epidural anesthesia, PCNL-GA = percutaneous nephrolithotomy under general anesthesia, PCNL-LIA = percutaneous nephrolithotomy under local infiltration anesthesia.

In all included studies, there were 6 studies^[14–19]^ comparing operative time. As for the operative time, PCNL-LIA offered a significantly shorter operative time compared with PCNL-GA or PCNL-CSEA for UUC (MD = −18.91, 95% CI: −26.47 to −11.35, *P*<.00001, *I*^2^ = 96%) (Fig. 4).

#### 3.2.3. Hospitalization expenses

**Figure 5.**
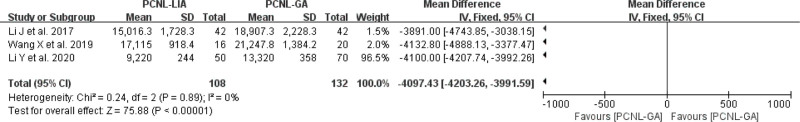
Forest plot comparing hospitalization expenses between PCNL-LIA and PCNL-GA. PCNL-GA = percutaneous nephrolithotomy under general anesthesia, PCNL-LIA = percutaneous nephrolithotomy under local infiltration anesthesia.

In all included studies, there were 3 studies^[14,15,18]^ comparing hospitalization expenses. Referring to the hospitalization expenses, PCNL-LIA offered a significantly lower hospitalization expenses compared with PCNL-GA for UUC (MD = −4097.43, 95% CI: −4203.26 to 3991.59, *P* < .00001, I^2^ = 0%) (Fig. 5).

#### 3.2.4. Complication rate

In all included studies, there were 4 studies^[13,14,17,18]^ comparing complication rate.

**Figure 6.**
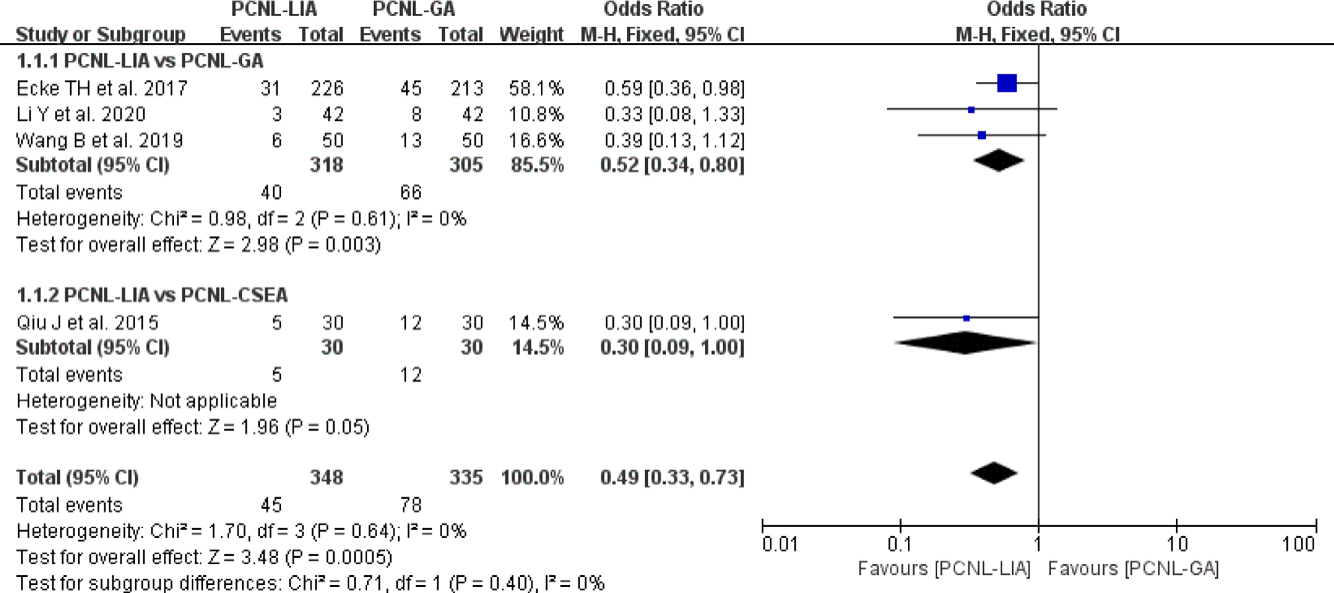
Forest plot comparing complication rate between PCNL-LIA and PCNL-GA or PCNL-CSEA. PCNL-CSEA = percutaneous nephrolithotomy under combined spinal-epidural anesthesia, PCNL-GA = percutaneous nephrolithotomy under general anesthesia, PCNL-LIA = percutaneous nephrolithotomy under local infiltration anesthesia.

Regarding the complication rate, PCNL-LIA provided a significantly lower complication rate compared with PCNL-GA or PCNL-CSEA for UUC (OR = 0.49, 95% CI: 0.33–0.73, *P* = .0005, *I*^2^ = 0%) (Fig. 6)

#### 3.2.5. Postoperative hospital stay

**Figure 7.**
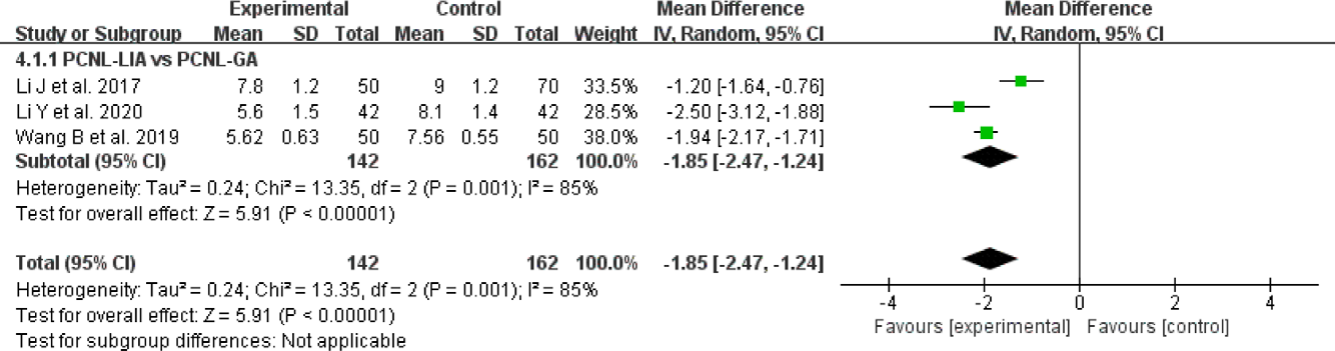
Forest plot comparing postoperative hospital stay between PCNL-LIA and PCNL-GA. PCNL-GA = percutaneous nephrolithotomy under general anesthesia, PCNL-LIA = percutaneous nephrolithotomy under local infiltration anesthesia.

In all included studies, there were 3 studies^[14,15,18]^ comparing postoperative hospital stay. Speaking of postoperative hospital stay, PCNL-LIA provided a significantly shorter postoperative hospital stay compared with PCNL-GA for UUC (MD = −1.85, 95% CI: −2.47 to 1.24, *P* = .001, *I*^2^ = 85%) (Fig. 7).

### 3.3. Sensitivity analysis

**Figure 8.**
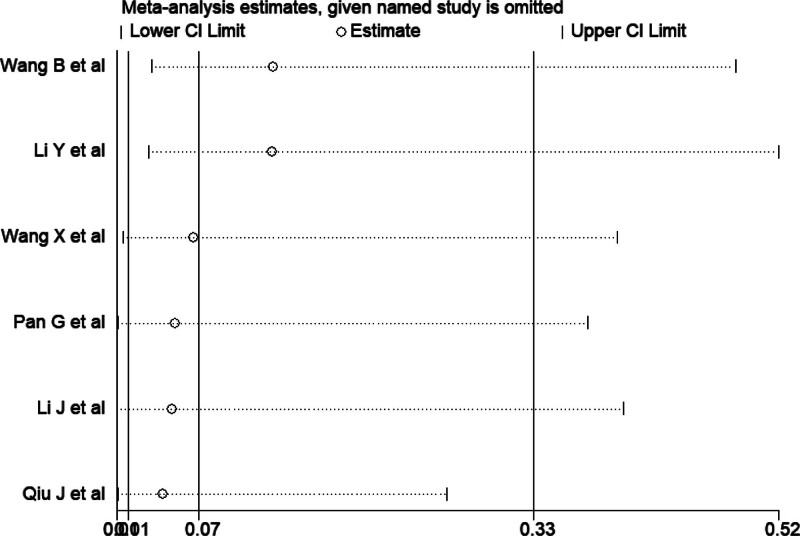
Sensitivity analysis.

To further assess the heterogeneity in these pooled studies, a sensitivity analysis was performed to thereby evaluate the influence of individual studies on the overall safety and efficacy of sonography-guided PCNL-LIA and PCNL-GA or PCNL-CSEA for UUC. Due to the small difference in scores of NOS and jadad among related studies, we did not consider adding weights to a particular study in sensitivity analysis to address potential biases. The results are shown in Figure 8. We found that when each 1 or each 2 trials were excluded, the heterogeneity has not yet decreased from high to the level that we could accept. Finally, the heterogeneity was significantly reduced (I^2^ = 0%) when 4 trials^[14,17–19]^ were excluded from the meta-analysis (Table 3). Consequently, after analyzing the existing data, we finally attributed the high heterogeneity to the difference in stone size, technical level of surgeons differences in medical level and economic levels in different regions and so on.

**Table 3:** Studies excluded.

Study	Step	*I*-squared
Wang et al 2019^[18]^	1	96.139582
Li et al.2020^[14]^	2	84.402584
Qiu et al.2015^[17]^	3	73.959286
Wang et al.2019^[19]^	4	0

### 3.4. Publication bias

**Figure 9.**
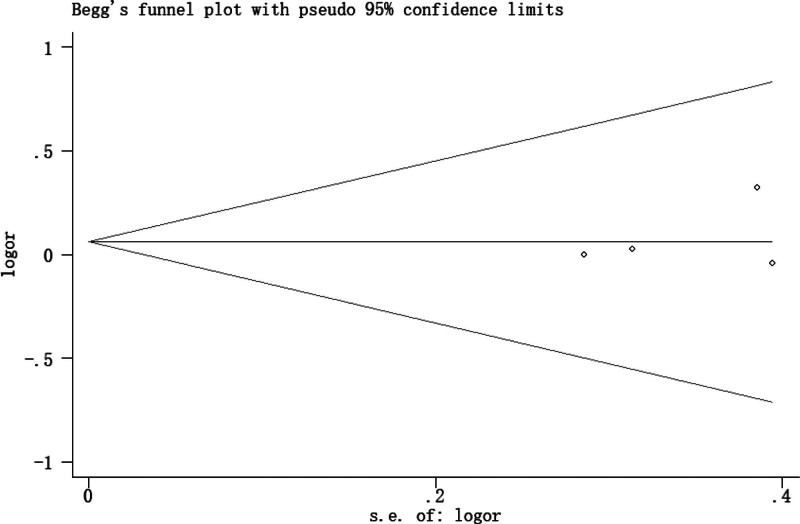
Funnel plot to detect publication bias (Begg test).

**Figure 10.**
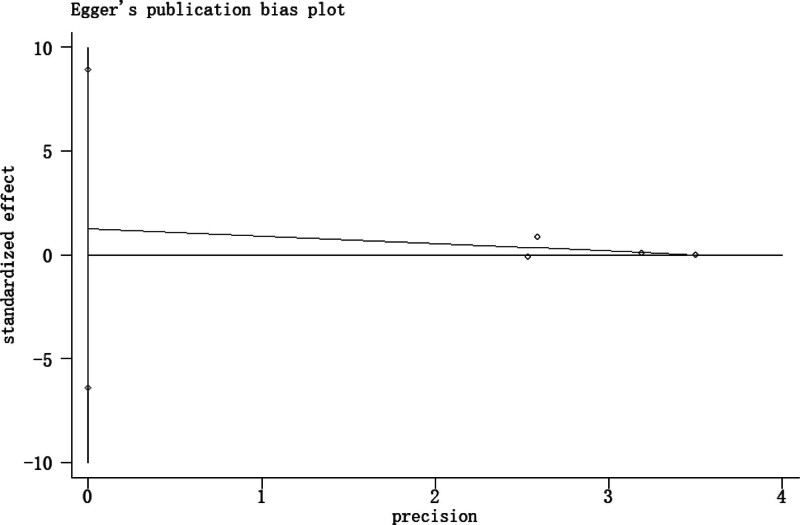
Funnel plot to detect publication bias (Egger test).

There was no evidence of publication bias to be observed by visual inspection of the funnel plots (Figs. 9 and 10) or by the application of Begg test (*P* = 1.0) or Egger test (*P* = .553).

## 4. Discussion

The present meta-analysis results demonstrated that both of them were safe and effective for patients of UUC. Our meta-analysis and systematic review is the first article, in many aspects, reporting on outcomes of comparing the safety and efficacy of sonography-guided PCNL-LIA and PCNL-GA or PCNL-CSEA for UUC. We identified 7 articles,^[13–19]^ that described the outcomes of sonography-guided PCNL-LIA and PCNL-GA or PCNL-CSEA for UUC. Although there are 7 of the included studies,^[13–19]^ whose quality scores, evaluated by the NOS and the Jadad Scale, were high or low, it is not influence decisions to pool studies in the meta-analysis. According to the meta-analysis results of synthesizing 7 included studies,^[13–19]^ involving 899 patients, we know that: when you choose a treatment for patients of UUC, comparing with PCNL-GA or PCNL-CSEA, PCNL-LIA offered a shorter operative time, lower hospitalization expenses, lower complication rate and shorter postoperative hospital stay for UUC.

For large renal stones (>2 cm), percutaneous nephrolithotomy (PCNL) is considered a first-choice treatment because of offering the best SFR compared with other modalities such as shock wave lithotripsy and retrograde intrarenal surgery (RIRS).^[20]^ PCNL is associated with postoperative complications that are more pronounced in frail and morbidly obese patients and patients with advanced chronic obstructive pulmonary disease.^[21–24]^ PCNL is usually performed under general anesthesia (GA) or CSEA due to the perceived risks of sudden patient movements and pain, resulting in pelvicaliceal system perforation or kidney parenchyma injury, and other advantages such as better control of breathing and better tolerability for patients.^[24–26]^ There are some disadvantages of GA such as pulmonary, vascular, and neurologic systems problems associated with electrolyte balance and the endotracheal tube especially during the change of position from lithotomy to prone.^[24,25]^ In recent years, several efforts have been attempted to explore the safety and feasibility of PCNL-LIA, because the side effects of GA itself might affect the outcomes of the surgery, such as in elderly patients, frail patients, and patients with severe chronic obstructive pulmonary disease.^[24]^ PCNL-LIA has the characteristics of rapid recovery, low cost and low requirements for patients’ cardiopulmonary function.^[15]^ Compared to PCNL-GA or PCNL-CSEA, PCN-LIA has no additional training requirements for surgeons.^[16]^ Thanks to the advantages of PCN-LIA, it is expected to be fully implemented in clinical practice in the future, which will reduce the economic cost and benefit patients. However, so far there is no meta-analysis comparing safety and efficacy of PCNL-LIA and PCNL-GA orPCNL-CSEA for UUC. Therefore, the present study focused on a comprehensive meta-analysis of existing evidence to quantify and compare the safety and efficacy of PCNL-LIA and PCNL-GA or PCNL-CSEA for UUC.

SFR occupied a key parameter in the process of estimating the efficacy of stone operation procedure.^[27,28]^ The study of Pan G et al^[16]^ considered that PCNL-LIA offered a higher SFR than PCNL-CSEA for UUC. Most studies^[14,17,18]^ thought that PCNL-LIA and PCNL-GA had no significant difference in SFR in the treatment of UUC. The results of this meta-analysis, like most studies,^[14,17,18]^ suggested that the stone clearance rate of PCNL-LIA and PCNL-GA in the treatment of UUC was similar. If the sample size of the study could be further increased, would different results be obtained? Unfortunately, there is currently a lack of more data, and it is hoped that there will be more and better studies in the future to support and validate it. For now, we could only think that the different results of SFR might be due to the difference in stone size and technical level of surgeons.

Li et al study^[14]^ included 120 patients showed that the operation time of PCNL-LIA was significantly shorter than PCNL-GA. And Li et al study^[15]^ included 84 patients also showed the same conclusion. But Qiu et al study^[17]^ included 60 patients showed that there was not much difference in the operation time between PCNL-LIA and PCNL-CSEA. Our study result showed that the operation time of PCNL-LIA was significantly shorter than PCNL-GA or PCNL-CSEA. The heterogeneity of operative time is high, and we have considered performing a subgroup analysis to identify the cause. However, due to the lack of specific data on stone size and patient comorbidity, we are unable to proceed further. In addition, regarding the subgroup analysis of research types, as there is only 1 retrospective case control in the studies that include the indicator of operative time, the heterogeneity of the subgroup analysis obtained in this way is still high. Finally, we hypothesize that the most likely reasons for this heterogeneity may be the surgeon’s surgical skills, stone size, and hardness, among others.

More research should be conducted to verify the correctness of these conclusions.

As for hospitalization expenses, because PCNL-LIA could save the cost of anesthetic drugs, the vast majority of research results^[14,15,19]^ indicated that the hospitalization cost of PCNL-LIA was significantly lower than that of PCNL-GA or PCNL-CSEA, which was still widely recognized. We compared data from 3 studies in total, all of which were conducted in China and used the RMB as their currency. There were no currency or country differences, and the results were reliable. Our research results have once again confirmed this point that the hospitalization cost of PCNL-LIA was significantly lower than that of PCNL-GA or PCNL-CSEA.

On the other hand, researchers have different views on complication rate. Some research^[13]^ thought that the complication rate of PCNL-LIA and PCNL-GA was similar. Some researchers^[14,18]^ have put forward inconsistent views, believing that the complication rate of PCNL-LIA is lower than that of PCNL-GA or PCNL-CSEA. Our research result was consistent with the latter. The complication rate was not only related to the operative methods, but also to the surgeon’s technique and experience, stone hardness, size and location, patient’s physical condition, duration of surgery, and whether other underlying diseases at present. However, PCNL-LIA does provide a new option for patients who cannot tolerate GA.

As for postoperative hospital stay, most people^[14,15]^ expressed the viewpoint that PCNL-LIA had the shorter hospital stay than PCNL-GA or PCNL-CSEA. This view was consistent with our results. This might be related to a series of views. PCNL-LIA had little impact on the physiological function of the elderly and rarely caused complications related to GA or epidural anesthesia.^[15]^ PCNL-LIA was simple, safe, effective, easy to master, and saved anesthesia time. There was no need for fasting before and after PCNL-LIA, which was conducive to the rapid recovery of patients, shortening the length of hospital stay and reducing the cost of hospitalization. The patients had a clear consciousness during the operation, and could timely feed back the intraoperative discomfort, which was conducive to the timely adjustment of surgical strategy. After PCNL-LIA, the patients waked up quickly without going to the recovery room for resuscitation, which reduced the postoperative intervention. As same as operative time, the heterogeneity of postoperative hospital stay is high, and we also have considered performing a subgroup analysis to identify the cause. But there are only 3 studies included in total, and conducting subgroup analysis is of little significance. Finally, we hypothesized that the most likely cause of this heterogeneity might be the patient’s physical condition, local medical health level. and so on Future research and data are needed to validate this theory.

There were some limitations in our meta-analysis. First, some missing data was not obtained despite repeated attempts to contact the authors. The potential impact of missing data (e.g., unpublished negative studies) or the lack of patient-reported outcomes (e.g., pain scores, satisfaction) will make the results biased. In the future, more data should be collected to verify the correctness of this conclusion. Second, the small number of studies (n = 7) limits the power of Begg and Egger tests. Third, there was bias in the country of the authors of included studies, although we used some measures to minimize it, it did not abolish them. We performed a sensitivity analysis to explain the high heterogeneity, but it could not descend to the degree that we could accept. It is a hypothesis of ours that there are several logical reasons such as stone size, surgeon expertise to explain the high heterogeneity among some studies, but the accuracy of our study was partly influenced. Future, better well-designed retrospective case controls and RCTs with high quality especially in some aspects such as larger sample sizes, standardized outcome definitions, or inclusion of diverse populations are needed. Yet another limitation, most of the studies included in this meta-analysis are from China, and due to differences in healthcare systems, surgical expertise, the applicability of the results is limited to some extent. In the future, more studies should be analyzed again to improve the applicability of the results to people all over the world.

## 5. Conclusions

This meta-analysis compared efficacy and safety of PCNL-LIA and PCNL-GA or PCNL-CSEA for UUC. Both of them were safe and effective for patients of UUC. Comparing with PCNL-GA or PCNL-CSEA, PCNL-LIA offered a shorter operative time, lower hospitalization expenses, lower complication rate and shorter postoperative hospital stay for UUC. Of course, PCNL-LIA may not be suitable for all patients (e.g., those with complex stones or requiring prolonged surgery). It is only a new treatment option for patients with UUC who cannot undergo PCNL-GA or PCNL-CSEA. Furthermore, more RCTs and retrospective case control studies are needed to certify these conclusions.

## Author contributions

**Conceptualization:** Junbo Liu, Yong Chen, Qiang Liu, Fei Lai.

**Data curation:** Junbo Liu, Qiang Liu, Fei Lai.

**Formal analysis:** Junbo Liu, Qiang Liu, Fei Lai.

**Methodology:** Yong Cai, Junbo Liu, Yong Chen, Qiang Liu, Fei Lai.

**Project administration:** Yong Cai.

**Resources:** Yong Chen.

**Software:** Yong Cai, Junbo Liu, Yong Chen, Fei Lai.

**Writing – original draft:** Junbo Liu.

**Writing – review & editing:** Yong Cai.
